# Supplementary material for: Cross-species imputation and comparison of single-cell transcriptomic profiles
Source: Genome Biol. 2025 Feb 26;26:40. doi: 10.1186/s13059-025-03493-x (PMC11863430; doi:10.1186/s13059-025-03493-x)
Supplement: Supplementary file 2 — Additional file 2. Review history. [file 13059_2025_3493_MOESM2_ESM.docx]

**1^st^ round**

**Reviewer 1**

Icebear is a deep learning model that is designed to integrate cross-species, single-cell profiles. Icebaer successfully removes species effects in the M1 dataset, shown via UMAP visualizations. Also, Icebear enhances its gene mapping by leveraging transitive relationships, creating a more comprehensive graph. This approach helps capture additional connections between genes, providing a better dataset for analysis. The paper goes into further analysis of prediction performance of functionally conserved genes (housekeeping) vs functionally diverged genes (non-housekeeping genes). In particular, the authors showcase the usefulness of Icebear in a relevant and impactful usecase by predicting the gene expression profiles in humans in a diseased state (AD) by training the model on gene expression profiles in mouse (healthy+diseased) and humans(healthy). The authors also demonstrate how evolutionary changes in regulation patterns of genes based on their position on the genome can be predicted and generalized. Overall, the applications demonstrated are interesting. However, we think the paper can be improved based on the following comments:

1. Icebear's reliance on 1-1 orthologs from Biomart, which may prioritize results based on sequence similarity, is a limitation that can lead to missing more implicit relationships between genes. This contrasts with methods like SATURN (https://www.nature.com/articles/s41592-024-02191-z), which utilizes Metagenes as a more comprehensive and elegant solution to capture many-many mappings based on functional relationships and sequence similarity. It would be worth testing the performance change by using mapping between metagenes.

2. As a cross-species analysis method, the datasets used have at most 3 species, which is a very small number of species. Infact, the cross species prediction is done on a dataset with only 2 species (mouse and human). A dataset with more than three species could be used to showcase Icebear's ability to handle multi-species analysis across a wider range of evolutionary distances.

3. Icebear evaluates cross-species prediction performance at a cell type level (pseudobulk profile), not the level of individual cells. This could be improved by finding closest cross-species neighbors for each cell and evaluating the model for each individual cell.

4. Incorporating a GAN into Icebear alters its architecture significantly. The authors could enhance their analysis by providing ablation test on the GAN model, with performance comparison between the model with and without the GAN.

5. There are other methods that perform cross-species prediction (like SAMap, SATURN). The paper does not provide performance comparison with any of these methods.

6. In the sci-RNA-Seq3 experiment, samples were processed in the same batch. Is it still considered that there is batch effect? Some discussion on under what cases one should model batch effect is needed.

7. In order to learn and separate the species factor, cell factor, and batch factor, does the model assume independence between these factors? If yes, it would be worth discussing the limitations of the model, as these factors may not be independent in reality.

8. The following points need clarification:

(1) Paper is not consistent in stating what exactly are the factors that gene expression is decomposed into. The mathematical definitions include tissue as one of the factors, but later the methods only consider species and batch. Maybe tissue type would be a good condition to incorporate if it is not already done. (2) Page 8, ll 57-60: What exactly is the celltype baseline? It is defined but what is the biological intuition? And how is this closeness measured? (3) If the genes that have changed from autosome to XCR/XAR are known, why do we need Icebear to predict gene levels on a cell by cell basis? The normalized cell type specific/tissue specific bulk profiles should also illustrate the same pattern. It would be interesting to compare the results obtained from these approaches to see if they align.

**Reviewer 2**

The manuscript "Cross-species imputation and comparison of single-cell transcriptomic profiles" describes a neural network-based model, IceBear, for comparing and predicting single-cell gene expression profiles across distantly related species. As a framework, it uses conditional variational autoencoders and one-to-one mapping of features (orthologous genes) between species. The model is validated on several atlas-scale scRNASeq datasets and applied to studying the evolution of X-linked genes and predicting changes in expression in a neurodegenerative disease (AD) from single cell data obtained in model organisms.

I find the study thoroughly designed, technically sound, and described with a sufficient level of detail. However, some aspects of novelty and impact remain unclear to me, which I detail below. I think these aspects must be clarified for a publication in Genome Biology. Additionally, the manuscript's language lacks clarity and rigor in many places.

1) Novelty of the deep learning methodology: Are there any significant advances in the presented approach compared to existing standard frameworks such as scVI, which is the basis for this study? The authors say it "is adapted" from scVI, but besides defining concrete label types for correcting species differences as a sort of sample-level batch type, the specific advance is not clear to me. The method description literally introduces a conditional variational autoencoder framework, which seems unnecessary. The role and necessity of adversarial learning remain unclear in the end.

2) The choice of baselines looks adequate, but I miss an intermediate baseline between the donor-based and species-based approaches. For example, a species-like baseline using a method from the single-cell data integration toolbox or a domain adaptation method.

3) Introducing one-to-one orthology mapping: How original is this methodological contribution? What are the fundamental limitations of this approach? How distant can the species be for such an analysis? To what extent does the evolution of ohnologs impact the results relying on establishing such a one-to-one mapping? Do I understand correctly that the idea of considering transitive relations allows resolving only a small fraction of new gene-gene relations, which can, in principle, be neglected?

4) The possibility of predicting gene expression changes in human tissues from model organisms looks very promising. However, the impact of such capability on clinical or evolutionary research remains superficially documented. Can the authors illustrate the application of the model to a clinically relevant case in studying AD? Predicting the increase or decrease of gene expression in XAR or XCR by the model looks convincing, but it remains unclear what existing challenging questions in evolutionary biology it answers. Is this approach applicable to understanding cancer, considering cancer cells "alien" to the organism (belonging to a related but different species)?

5) The data used for the study come from profiling specific organs (brain and heart) characterized by specific properties of cell type diversity and composition. To what extent does this impact the conclusions? I appreciate that a truly comparative analysis might be impossible here, but some discussion seems appropriate.

6) The authors report that cross-species housekeeping gene expression can be relatively reliably predicted. Housekeeping genes are expected to have higher expression as well independently of their evolution, hence their expression estimation is expected to be more reliable. Can this be checked? Can insights be obtained for the genes with the worst predictions (in other words, can we split non-housekeeping genes into meaningful subcategories with different prediction levels)?

7) I did not find a reference to the IceBear codebase (including ortholog mapping) anywhere in the text. I expect the code to be publicly available and documented, and the computational results to be reproducible. I would like to be able to assess the code before taking a final decision on publication.

8) Is it envisageable/meaningful to consider alternative VAE architectures for this task, e.g., using coupled VAEs, considering different species as different modalities, and the problem as a language translation problem?

Some minor points:

1) Is the median sequencing depth of 450 UMIs considered enough for studying single-cell level expression of over 10,000 genes?

2) The sentence "This process yielded 10,030 genes with orthologs shared across the three species, 70 of which were added through transitive relationships," is repeated twice.

3) The definition of the "indiv_predictions" baseline remains unclear to me (what "the mean of similarity measurements between our predictions with each donor in the test set" exactly means). Overall, the description of the baselines lacks clarity; better to have more formal definitions.

4) Figure 2D is not informative and it is not clear how much it adds to the main text compared to Figure 2C. I suggest moving it to the supplementary materials.

5) What is Oligo-OPC? Any insight into why IceBear works worst for this cell type?

6) I lack an understanding of what the term "translation algorithm" means in the phrase "may not only follow a general cross-species translation algorithm."

7) Figure 2: "profiles in missing major cell types in the human motor cortex" : what does "missing" mean here? Held out?

8) Multiple unclear formulations: just a couple of examples: "log2 fold change of genes across species," "asking whether gene expression increases from halved gene dose when autosomal genes become X-linked and only have a single copy in XY males during mammalian sex chromosome evolution," etc.

9) Figures 4A and 4B—comparing them by visual inspection is not informative (especially considering the point sizes). Can this be quantified?

**Authors’ response**

We thank the reviewers for their positive assessment of our work and valuable suggestions. We have addressed all comments below.

Reviewer 1

Icebear is a deep learning model that is designed to integrate cross-species, single-cell profiles. Icebaer successfully removes species effects in the M1 dataset, shown via UMAP visualizations. Also, Icebear enhances its gene mapping by leveraging transitive relationships, creating a more comprehensive graph. This approach helps capture additional connections between genes, providing a better dataset for analysis. The paper goes into further analysis of prediction performance of functionally conserved genes (housekeeping) vs functionally diverged genes (non-housekeeping genes). In particular, the authors showcase the usefulness of Icebear in a relevant and impactful usecase by predicting the gene expression profiles in humans in a diseased state (AD) by training the model on gene expression profiles in mouse (healthy+diseased) and humans(healthy). The authors also demonstrate how evolutionary changes in regulation patterns of genes based on their position on the genome can be predicted and generalized. Overall, the applications demonstrated are interesting. However, we think the paper can be improved based on the following comments:

1. Icebear’s reliance on 1-1 orthologs from Biomart, which may prioritize results based on sequence similarity, is a limitation that can lead to missing more implicit relationships between genes. This contrasts with methods like SATURN, which utilizes Metagenes as a more comprehensive and elegant solution to capture many-many mappings based on functional relationships and sequence similarity. It would be worth testing the performance change by using mapping between metagenes.

We thank the reviewer for this valuable suggestion. One of the key strengths of Icebear is its ability to predict and assess how a gene’s orthologs change expression across species. Thus, we focus on one-to-one ortholog mapping with the purpose of directly swapping species factors to measure gene expression changes for orthologous genes during evolution. SATURN uses macrogenes to perform cross-species cell alignment but not expression changes, and by design it cannot predict or compare gene expression across species. In response to the reviewer’s suggestions, we have now compared Icebear with SATURN with respect to cross-species alignment, where we used the LISI score to measure, for each cell type, how well cells from different species are aligned. Icebear outperforms SATURN when testing cell alignment on the motor cortex dataset (Figure 1A–B in this document). For the cell-atlas dataset, Icebear performs comparably to SATURN (Figure 1C–D in this document). One possible reason for this difference in relative performance is that the cell atlas dataset has a much lower median UMI (mouse: 688; human: 591) compared to the motor cortex dataset (mouse: 12059.5; human: 20056), and by focusing on genes with one-to-one matching, our advantage over SATURN shrinks, as the reviewer suggested. We have added the above comparison to the results and supplementary materials and also suggested future directions to address this issue.

In main text:

To further quantify the performance of cross-species cell type matching, we compared Icebear with SATURN, a state-of-the-art cross-species alignment method [1]. Although cell type matching is only a by-product of Icebear, we find that Icebear can outperform or perform comparably to SATURN in this task (Supplementary Figure 1).

In supplement:

Figure 1: Icebear is comparable to or more accurate than SATURN at aligning single cells across species. (A) UMAP of SATURN-aligned cell embeddings of the motor cortex dataset, colored by the species each cell comes from. Median UMI across cells: mouse = 12060; human = 20056. (B) Boxplot distributions of LISI scores measuring how well mouse and human cells from the motor cortex dataset are aligned per cell type. All hyperparameters tuned are shown. (C) Similar to (A), UMAP of SATURN-aligned cell embeddings of the cell atlas dataset, colored by the species each cell comes from. Median UMI across cells: mouse = 688; human = 591. (D) Similar to (B), the LISI score distribution of cross-species alignment per cell type is measured on the cell atlas dataset.

1.1 Comparison with SATURN

One of the key strengths of Icebear is its ability to predict and assess how a gene’s orthologs change expression across species. In particular, by making use of one-to-one ortholog mapping, Icebear can directly swap species factors to measure gene expression changes for orthologous genes during evolution. In contrast, SATURN uses macrogenes to perform cross-species cell alignment but not expression changes, and by design is incapable of predicting gene expression changes across species [1].

To compare Icebear with SATURN, we therefore can only evaluate the two methods with respect to cross-species alignment. To do so, we trained SATURN and Icebear separately on the primary motor cortex (M1) dataset [2] and human and mouse cell atlases [3, 4], because these two datasets have matching cell-type annotations across species. For each method, we used the LISI score to measure, for each cell type, how well cells from different species are aligned. The SATURN paper adjusts a single hyperparameter, the number of macrogenes, so we experimented with values of 1000, 2000, 3000, and 4000. At 5000 macrogenes, SATURN exceeded the available GPU memory (8 GB).

In discussion:

Currently, Icebear is designed to model cross-species gene expression using one-to-one orthology relationships. This approach discards a subset of duplicated genes that potentially harbor valuable information about cellular function and evolution. In this study, we observe that the advantage of Icebear’s cross-species alignment performance against SATURN shrinks as the median cell UMI decreases (Supplementary Figure 1). In the future, inspired by ideas from SATURN [1] and coupled variational autoencoders [5], we could extend Icebear to include all genes, rather than only one-to-one orthologs. In principle, such an extension to Icebear could enhance the cross-species translation performance without losing information from non-orthologous genes.

2. As a cross-species analysis method, the datasets used have at most 3 species, which is a very small number of species. In fact, the cross species prediction is done on a dataset with only 2 species (mouse and human). A dataset with more than three species could be used to showcase Icebear’s ability to handle multi-species analysis across a wider range of evolutionary distances.

We focused on the current datasets for the following reasons. First, in many cases, researchers studying certain model organisms may be interested in transferring that knowledge to humans; thus, two species are involved. Second, another potential use case for Icebear is to predict how a gene’s expression would change from one species to another. Demonstrating the feasibility of solving this problem in the three-species setting suggests that it can, in principle, be generalized to more than three species through pairwise comparisons combinations of different species. Third, to compare gene expression across species, we need shared batch effects from single cells collected across species to attribute the cellular profile changes to biological variations instead of batch effects. We were unable to find datasets with more than three species sequenced from the same or overlapping batches to derive cross-species gene expression changes directly. We have performed additional analysis below:

In main text:

We further applied Icebear to concatenated datasets of five species generated across four studies (Supplementary Methods 1.3). Icebear is able to align cells across the five species (Supplementary Figure 2A). Furthermore, Icebear outperforms all baselines when predicting human cell typespecific profiles from cells in the frog and zebrafish (Supplementary Figure 2B). These results further demonstrate Icebear’s ability to generalize to multiple species across various evolutionary distances.

In supplement:

1.3 Multi-species evaluation

To address the question of how Icebear works on more than three species and evolutionarily distant species, we constructed a five-species dataset, consisting of our in-house generated heart dataset collected from mouse and chicken, along with other public heart datasets from human [6]), frog (Xenopus laevis) [7] and zebrafish (Danio rerio) [8]. No shared batches exist across these four datasets, making it infeasible to perform cross-species comparison.

To test cross-species alignment and prediction, we first performed one-to-one orthology mapping following the protocol described in the manuscript (Methods 2.3). The frog dataset separately measures genes from the L (long) and S (short) homologous chromosome sets. To approximate one-to-one orthology mapping, we took the sum of gene expression counts across the two sets of chromosomes for each gene. Overall, 8675 ortholog groups were mapped across all five species. Meanwhile, without the transitive mapping step, only 7697 genes were included. We then retained orthologs with measurements across all five species and only retained the biggest batch from the human, frog, and zebrafish datasets. Next, we filtered out genes expressed in fewer than 50 cells and cells with fewer than 100 genes expressed. The above process ends up with 3064 genes and 36,227 cells.

Finally, we trained the Icebear model on this five-species dataset. In the first task, we used the default hyperparameters by setting the dimension of the embedding layer to 25 and ignoring the discriminator step, and then training Icebear on all cells. Using the resulting model, we asked whether cells from different species are aligned to the same space. As a control, we also generated cell embeddings derived from UMAP on the original measurement. In the second task, we identified shared cell type labels from species where cell type annotation is available (i.e., human, frog, and zebrafish), held out each cell type in human, and trained an Icebear model on the rest of the cells. We report the Pearson correlation between the original held-out profile and Icebear-predicted human cell type-specific profile based on the corresponding cell type in the frog or zebrafish (Methods 2.6).

3. Icebear evaluates cross-species prediction performance at a cell type level (pseudobulk profile), not the level of individual cells. This could be improved by finding closest cross-species neighbors for each cell and evaluating the model for each individual cell.

Icebear can certainly make predictions at the single-cell level, but evaluating the quality of such predictions is challenging. The problem with the suggested approach of evaluating the model on closest cross-species neighbors is that there is no guarantee that such neighbors represent true cell correspondence across species. Because of this difficulty, in the paper we evaluated Icebear using high-resolution cell type annotation as well as via correspondences generated by prior biological knowledge.

4. Incorporating a GAN into Icebear alters its architecture significantly. The authors could enhance their analysis by providing ablation test on the GAN model, with performance comparison between the model with and without the GAN.

We thank the reviewer for the great suggestion. We have now performed an ablation study and compared the pseudobulk Pearson correlation between the predicted and original profile in the motor cortex dataset. We found that the discriminator does not significantly improve performance. Given that, we decided to provide the discriminator as an option (i.e., hyperparameter) to Icebear, and we changed the main text accordingly.

Figure 2: Icebear can be applied to multiple species. (A) UMAP of cell embeddings based on the original experimental measurement, cell embeddings are derived jointly and plotted separately across species.

(B) UMAP of cell embeddings based on the Icebear model, cell embeddings are derived jointly and plotted separately across species. (C) Barplot showing Pearson correlation coefficient (cor) between cross-species predicted and observed gene expression profiles in the “held-out” major cell types in human heart. For each cell type, the Pearson correlation coefficient is compared between Icebear’s prediction (dark green bar) and two baselines (species and celltype). Human profiles are either predicted based on cells in the frog (left) or zebrafish (right).

Figure 3: Ablation test on the discriminator model. Pseudobulk Pearson correlation between the predicted and original profile in the motor cortex dataset. Each dot indicates a cell type, colored by the major cell type annotation. The x-axis represents results generated by the Icebear model without the discriminator, and the y-axis shows results generated by Icebear with the discriminator as a hyperparameter. Numbers indicate the number of cell types above and below the diagonal. A p-value is calculated using Wilcoxon onesided signed rank tests under the null hypothesis that the median performance difference between Icebear with and without the discriminator is less than or equal to 0.

An ablation study comparing the pseudobulk Pearson correlation between the predicted and original profile in the motor cortex dataset found that including the discriminator does not significantly improve Icebear’s performance (Supplementary Figure 3).

5. There are other methods that perform cross-species prediction (like SAMap, SATURN). The paper does not provide performance comparison with any of these methods.

One unique contribution of Icebear is that it can directly perform cross-species prediction and comparison. Meanwhile, SAMap and SATURN focus on cross-species cell alignment. To address the reviewer’s comment, we compared Icebear’s cross-species alignment with SATURN and showed SATURN is no better than Icebear at cross-species alignment (see item 1 for details). Because SATURN has previously been shown to outperform SAMap and other existing methods, we did not further compare Icebear with other methods. In addition, we have now clarified the unique use case of Icebear in the introduction:

Although multiple methods have been developed that focus on aligning single cells across species [9, 10, 11, 1], these methods are not able to make cross-species comparison at single-cell resolution, nor can they make predictions about evolutionary changes when certain experimental measurements (e.g., a particular cell or tissue type of a species) are not available.

6. In the sci-RNA-Seq3 experiment, samples were processed in the same batch. Is it still considered that there is batch effect? Some discussion on under what cases one should model batch effect is needed.

The sci-RNA-Seq3 experiment consists of three batches (see Table 1). Thus, the Icebear model trained on this dataset has three types of batch factors, and cells belonging to the same batch are appended with the same batch factor during training. We have now clarified when batch effects are modeled, together with other factors, in the main text.

Our model reconstructs each scRNA-seq profile using a set of factors representing cell identity, tissue, species, and batch. The batch factor represents any knowledge of potential technical confounders, including studies, sequencing machines, or data generation batches. In the absence of prior knowledge of experimental batches, the batch factor is the same across all cells. Similarly, the tissue factor is kept constant if all cells are extracted from the same tissue type. These factors are concatenated and fed into a neural network to reconstruct the original gene expression profile. The model adapts a conditional variational autoencoder framework [9]. Species, tissue, and batch factors are encoded using one-hot encoding, while the model learns cell factors as n-dimensional vectors, where n is a hyperparameter to be tuned.

7. In order to learn and separate the species factor, cell factor, and batch factor, does the model assume independence between these factors? If yes, it would be worth discussing the limitations of the model, as these factors may not be independent in reality.

We agree, and we have added a discussion of this point:

Icebear assumes the cell factor is conditionally independent of other factors (such as species and batch), according to the biological knowledge that cell identities are not supposed to vary a lot across batches or species. This assumption allows us to swap factors such as species to directly assess how the same cell’s expression would change across species. Note that in some instances cell populations may be distinct across species (e.g., mice have tails and humans don’t), which will not fit into our model.

Beyond that, the model does not assume any independence, and in fact, we expect each cell’s gene expression profile to be the nonlinear combinatorial outcome of all factors. Icebear uses nonlinear neural network layers in the decoder to model such effects and reconstruct the cellular profile from the interaction effects among these factors.

8. The following points need clarification: (1) Paper is not consistent in stating what exactly are the factors that gene expression is decomposed into. The mathematical definitions include tissue as one of the factors, but later the methods only consider species and batch. Maybe tissue type would be a good condition to incorporate if it is not already done.

We thank the reviewer for catching this typo. We have tried adding tissue as a separate factor when training on the cell atlas dataset and found that adding a tissue factor further improves the performance (Figure 4 in this document).

We have therefore updated the results in Figure 3A (Figure 5 in this document) and corrected this point in the method and result section.

In methods:

Our model reconstructs each scRNA-seq profile using a set of factors representing cell identity, tissue, species, and batch. The batch factor represents any knowledge of potential technical confounders, including studies, sequencing machines, or data generation batches. In the absence of prior knowledge of experimental batches, the batch factor is the same across all cells. Similarly, the tissue factor is kept constant if all cells are extracted from the same tissue type. These factors are concatenated and fed into a neural network to reconstruct the original gene expression profile. The model adapts a conditional variational autoencoder framework [9]. Species, tissue, and batch factors are encoded using one-hot encoding, while the model learns cell factors as n-dimensional vectors, where n is a hyperparameter to be tuned.

Figure 4: Adding a tissue factor improves Icebear’s performance. Pseudobulk Pearson correlation between the predicted and original profile in the cell atlas dataset. Each dot indicates a cell type, colored by the tissue it is extracted from. The x-axis represents results are generated by the Icebear model without the tissue factor, and the y-axis shows results are generated by Icebear with a tissue factor. Numbers indicate the number of cell types above and below the diagonal. A p-value is calculated using Wilcoxon one-sided signed rank tests under the null hypothesis that the median performance differences between tissue-aware and tissue-agnostic Icebear model is less than or equal to 0.

Figure 5: Updated cross-species prediction performance on the cell atlas dataset. Barplot showing Pearson correlation coefficient (cor) between predicted and observed gene expression profiles in each heldout cell type per tissue. The Pearson correlation coefficient is compared for each cell type between Icebear’s prediction (dark green bar) and the species and cell type baselines. We also compared “indiv donorbaseline” (correlation between individual donors) with Icebear’s correlation with individual donors (“indiv prediction”, light green bar).

In results:

Also, explicitly modeling tissue as a factor significantly improves the cross-species prediction performance (Supplementary Figure 4).

(2) Page 8, ll 57-60: What exactly is the celltype baseline? It is defined but what is the biological intuition?

And how is this closeness measured?

An intuitive guess of the missing cell type’s profile in human is to approximate it as the profile from an existing cell type most similar to the missing one. In this way, especially when the cell types are defined at high resolution (e.g., the 45 cell types in the motor cortex dataset), we will be able to capture most characteristics of the missing cell type based on a similar existing cell type, making this a hard baseline to beat. Thus, the rationale behind the celltype baseline is to ensure that our prediction captures more meaningful signals of the missing cell type than guesses using a similar existing one. We have added this clarification to the method section.

1. Donor baseline (“donor baseline”): a “cheating” baseline that calculates the mean similarity across donors within the held-out test set. Here, we calculate the Pearson correlation of sequencing-depth normalized and log-transformed pseudobulk cell-type specific profiles between each pair of donors and report the mean correlation across all donor pairs.

2. Species baseline (“species baseline”): similarity between the original held-out cell type’s profile in human and the corresponding cell type’s profile in mouse. Genes are transferred to the target species through ortholog mapping and Pearson correlation between human and mouse sequencing-depth normalized and log-transformed pseudobulk cell-type specific profiles are reported.

3. Celltype baseline (“celltype baseline”): a “lazy” but close baseline that calculates the similarity between profiles of the held-out cell type and a similar cell type that has measurements in the target species. We first identify the cell type that has the most similar pseudobulk gene expression profile to the held-out cell type in the source species, calculated based on Euclidean distance on the sequencing-depth-normalized and log-transformed pseudobulk profiles. This baseline is created to ensure that our prediction captures more meaningful signals of the held-out cell type compared to guesses using a similar existing one.

(3) If the genes that have changed from autosome to XCR/XAR are known, why do we need Icebear to predict gene levels on a cell by cell basis? The normalized cell type specific/tissue specific bulk profiles should also illustrate the same pattern. It would be interesting to compare the results obtained from these approaches to see if they align.

We thank the reviewer for bringing up this important question. It should be noted that there has been some controversy in the literature about the upregulation of X-linked genes when they changed from autosomes to the X chromosome regions XCR and XAR, which Icebear helps clarify. We now present the analysis using pseudobulk profiles for a comparison (Figure 6 in this document) and explain the advantages of Icebear in the discussion.

Figure 6: Comparison of XCU patterns between Icebear and pseudobulk measurements (A) Boxplot of log2 fold change of genes across species predicted by Icebear, with genes grouped by their Xlinked pattern. The statistical significance of XCU events is calculated using the Wilcoxon one-sample rank sum test and subjected to multiple hypothesis correction. (B) Similar to (A), except that the log2 fold change patterns are calculated based on pseudobulk gene expression measurements. (C) Gene copy number changes in males across species.

Icebear has the following advantages when compared with pseudobulk-based comparison (Supplementary Figure 6). First, Icbear can discover XCR/XAR patterns when gene expression measurements are missing in certain species and tissues; i.e., we are able to predict the X chromosome upregulation pattern for the heart when there are no measurements available in the opossum heart. Second, Icebear’s cross-species comparison does not require any known matching of cell type labels across species or assumption of an equal proportion of cell type compositions between species, which greatly broadens the use cases where there are no existing matched cell type annotations across species. Third, comparing our prediction with the original measurements, we found general agreement of X chromosome up-regulation (XCU) patterns inferred from Icebear and pseudobulk profiles. However, XCU patterns are more apparent by Icebear imputation (as shown by increased significance) than simple pseudobulk comparison, because the latter may suffer from insufficient normalization cross-species and complex cell type composition. In addition, Icebear can predict XCU events when genes in the XAR region moved from the chicken autosome to the mouse X crhomsome, whereas the pseudobulk-based measurements cannot, again suggesting the sensitivity of Icebear. Icebear’s prediction agrees with the previous knowledge that mouse X-linked genes are subject to XCU and also suggests for the first time that the extent of XCU varies between regions with different evolutionary origins.

Reviewer #2

The manuscript “Cross-species imputation and comparison of single-cell transcriptomic profiles” describes a neural network-based model, IceBear, for comparing and predicting single-cell gene expression profiles across distantly related species. As a framework, it uses conditional variational autoencoders and one-toone mapping of features (orthologous genes) between species. The model is validated on several atlas-scale scRNASeq datasets and applied to studying the evolution of X-linked genes and predicting changes in expression in a neurodegenerative disease (AD) from single cell data obtained in model organisms. I find the study thoroughly designed, technically sound, and described with a sufficient level of detail. However, some aspects of novelty and impact remain unclear to me, which I detail below. I think these aspects must be clarified for a publication in Genome Biology. Additionally, the manuscript’s language lacks clarity and rigor in many places. 1) Novelty of the deep learning methodology: Are there any significant advances in the presented approach compared to existing standard frameworks such as scVI, which is the basis for this study? The authors say it “is adapted” from scVI, but besides defining concrete label types for correcting species differences as a sort of sample-level batch type, the specific advance is not clear to me. The method description literally introduces a conditional variational autoencoder framework, which seems unnecessary. The role and necessity of adversarial learning remain unclear in the end.

We thank the reviewer for this suggestion, and we have added clarifications to the main text accordingly. The major difference between Icebear and scVI is that scVI focuses on probabilistic modeling of existing singlecell measurements but not on inferring missing single-cell profiles in another species or cell type. The other novelty is that Icebear can directly make gene expression comparisons at cellular resolution across species. Besides that, to make sure that gene expression values are directly comparable across species, Icebear does not perform variational inference of the sequencing depth factor but instead directly corrects for the effect of sequencing depth in the zero-inflated negative binomial approximation step, and then uses housekeeping genes to anchor cross-species expression comparison.

Although multiple methods have been developed that focus on aligning single cells across species [9, 10, 11, 1], these methods are not able to make cross-species comparison at single-cell resolution, nor can they make predictions about evolutionary changes when certain experimental measurements (e.g., a particular cell or tissue type of a species) are not available.

Regarding adversarial learning, we did an ablation study and found that, indeed, it is not critical (see item 4 above). We thank the reviewer for the great suggestion and have thus decided to provide the discriminator as a hyperparameter to tune for.

2) The choice of baselines looks adequate, but I miss an intermediate baseline between the donor-based and species-based approaches. For example, a species-like baseline using a method from the single-cell data integration toolbox or a domain adaptation method.

We have not figured out a way to create an intermediate baseline between the donor- and species-based approaches. Regarding the suggestion of creating a species-like baseline using a single-cell data integration toolbox, we came up with a more strict baseline on top of a state-of-the-art single-cell integration tool and demonstrate that Icebear significantly outperforms such a baseline at recapitulating held-out human profiles using Pearson correlation (Figure 7 in this document).

In main text:

To further quantify the performance of cross-species cell type profile prediction, we created another baseline that is based on cross-species alignment [10] followed by k-nearest-neighbor calculation, and we found that Icebear significantly outperforms this baseline (Supplementary Figure 7). Figure 7: Comparison of Icebear and Harmony+kNN baseline Pseudobulk Pearson correlation between the predicted and original profile in the mouse motor cortex dataset. Each dot indicates a cell type, colored by the major cell type annotation. The x-axis represents results generated by the Harmony+kNN baseline, and the y-axis shows results generated by Icebear. Numbers indicate the number of cell types off-diagonal. A p-value is calculated using Wilcoxon one-sided signed rank tests.

In supplementary methods section:

1.2 Comparison with Harmony+kNN

We repeat the same protocol and evaluation of cell type prediction in the motor cortex dataset, where we hold out a cell type in human one at a time, run Harmony [10] to align the rest of the cells across species, retrieve the k-nearest human cells of each mouse cell from the held-out cell type, and use the pseudobulk profiles of all the k-nearest human cells as the baseline prediction. We tuned twice as many hyperparameters (12, rather than 6) for this method, including the number of principal components (PCs) used for Harmony (n=25, 50, 100), and the number of neighboring cells used to make the prediction (k=5, 20, 80, 320). To select the best hyperparameters, we first choose the number of PCs based on cross-species cell alignment (calculated by the LISI score). We then select the best k based on a “cheating“ method, where we pretend we already know the held-out cell type’s nearest neighboring cell type correspondingly in human and mouse, and we select the best model that gives the best pseudobulk Pearson correlation prediction on the neighboring cell type. This method creates an unfair baseline for Icebear, as Icebear does not leverage any cell type information during the training or model selection stages.

3) Introducing one-to-one orthology mapping: How original is this methodological contribution? What are the fundamental limitations of this approach? How distant can the species be for such an analysis? To what extent does the evolution of orthologs impact the results relying on establishing such a one-to-one mapping? Do I understand correctly that the idea of considering transitive relations allows resolving only a small fraction of new gene-gene relations, which can, in principle, be neglected?

The orthology mapping adopts the general idea of finding pairwise matches with the highest confidence, then proposes a greedy algorithm to fill in transitivity relationships. To our knowledge, this approach is indeed novel, but we do not intend this to be a key methodological contribution of our work. We developed this approach because it seemed like the most straightforward way to solve the problem at hand. To address the importance of transitive relationships, we performed an additional ortholog mapping across five species (i.e., human, mouse, chicken, frog, and zebrafish). This time, we observed 8675 orthology matches across all five species, whereas without the transitive mapping step, only 7697 orthology matches are retained. As the reviewer pointed out, the major limitation is that we only focus on genes with known ortholog relationships and lose genes without one-to-one orthology mapping. We have added this point to the discussion below.

Currently, Icebear is designed to model cross-species gene expression using one-to-one orthology relationships. This approach discards a subset of duplicated genes that potentially harbor valuable information about cellular function and evolution. In this study, we observe that the advantage of Icebear’s cross-species alignment performance against SATURN shrinks as the median cell UMI decreases (Supplementary Figure 1). In the future, inspired by ideas from SATURN [1] and coupled variational autoencoders [5], we could extend Icebear to include all genes, rather than only one-to-one orthologs. In principle, such an extension to Icebear could enhance the cross-species translation performance without losing information from non-orthologous genes.

4) The possibility of predicting gene expression changes in human tissues from model organisms looks very promising. However, the impact of such capability on clinical or evolutionary research remains superficially documented. Can the authors illustrate the application of the model to a clinically relevant case in studying AD? Predicting the increase or decrease of gene expression in XAR or XCR by the model looks convincing, but it remains unclear what existing challenging questions in evolutionary biology it answers. Is this approach applicable to understanding cancer, considering cancer cells “alien” to the organism (belonging to a related but different species)?

We thank the reviewer for suggesting more discussion on the clinical and evolutionary capacity of Icebea.

We have added the following discussions:

In evolutionary biology, it is still controversial whether there XCU occurs in mammals and, if so, what and how X-linked genes are upregulated during sex-chromosome evolution. The challenge is the lack of appropriate data and computational methods to directly compare expression levels of orthologous genes before and after changing from autosomal to X-linked. Understanding Xchromosome dosage regulation is important to understand the impact of large-scale gene dosage alterations such as chromosome aneuploidy, which are often linked to cancer and developmental disorders. One of the major contributions of Icebear is its ability to compare gene expression changes across species at cellular resolution, so that we can reveal how orthologous gene expression evolves across different cells and tissues. In this work, we demonstrated that up-regulation of X-linked genes occurs in an evolutionary and diverse pattern and is, at least partially, associated with enhancing RNA stability. These findings provide new evolutionary insights into the time and mechanisms of XCU. In addition, by looking at the differences between the actual gene expression and the predicted value across species, we have observed the cell types and genes that have the most evolutionary divergence, which have the potential to explain what cell types and genes are conserved across species or adapted to species-specific needs. In this study, we demonstrated as a proof of principle how we can predict human AD profiles based on mouse AD models. We envision this method being applied to different diseases, such as Rett syndrome, Parkinson, lupus, or progeria where human samples (e.g. brain) are difficult to obtain.

The idea of applying Icebear to cancer studies is exciting, though cancer cells often stem from one cell with mutations accumulating across time, so they may not have a clear population-wise correspondence to normal cells for Icebear to recover the pattern.

5) The data used for the study come from profiling specific organs (brain and heart) characterized by specific properties of cell type diversity and composition. To what extent does this impact the conclusions? I appreciate that a truly comparative analysis might be impossible here, but some discussion seems appropriate.

We have clarified that Icebear can generalize to different organs and cell type compositions in the following discussion. Given this rationale, we performed single-cell cross-species inference for all cells in the brain and heart as a proof-of-principle study to demonstrate that Icebear can reveal meaningful cross-species gene expression changes.

In our analysis, we have tested Icebear on both an atlas-level dataset covering 12 organs and 9 major cell types [3, 4], and a domain-specific dataset on motor cortex covering 10 major cell types and 45 fine-grained cell types [2]. Our results suggest that Icebear can perform accurate single-cell cross-species inference across various tissues and cell types.

6) The authors report that cross-species housekeeping gene expression can be relatively reliably predicted. Housekeeping genes are expected to have higher expression as well independently of their evolution, hence their expression estimation is expected to be more reliable. Can this be checked? Can insights be obtained for the genes with the worst predictions (in other words, can we split non-housekeeping genes into meaningful subcategories with different prediction levels)?

We thank the reviewer for this great suggestion. We have performed additional analyses based on this suggestion and added them to the manuscript (Figure 8 and Figure 9 in this document).

To test whether the better predictive performance of housekeeping genes is due to higher gene expression, we divided all genes into 10 equal-sized bins based on pseudobulk gene expression across all cells and compared the predictive error of housekeeping versus nonhousekeeping genes within each expression bin (Supplementary Figure 8). The result suggests that genes with lower expression are likely to have a higher predictive error, and housekeeping genes tend to have lower predictive error than their expression-matched non-housekeeping genes. To gain insights into what factors drive such differences, we further hypothesized that genes with high expression variation across cells may be more challenging to predict than genes with relatively consistent expression. To test the hypothesis, we binned genes by expression variation across cells (Supplementary Figure 9). Intriguingly, while variance contributes to some performance differences, housekeeping genes still tend to have lower relative prediction errors than non-housekeeping genes. The above evidence suggests that Icebear’s prediction performance may reflect meaningful biological changes during evolution.

7) I did not find a reference to the IceBear codebase (including ortholog mapping) anywhere in the text. I expect the code to be publicly available and documented, and the computational results to be reproducible. I would like to be able to assess the code before taking a final decision on publication.

We apologize for this oversight. We have added the GitHub link to the manuscript.

Figure 8: Relative prediction error across cell types and levels of pseudobulk gene expression Boxplots of genes relative prediction error across cell types. Genes are separated by their properties, and ten equal-sized expression bins are sorted from lowest (bin 1) to highest (bin 10).

Figure 9: Relative prediction error across cell types and levels of gene expression dispersions Boxplots of genes relative prediction error across cell types. Genes are separated by their properties and ten equal-sized bins based on their expression dispersion across all cells, from lowest (bin 1) to highest (bin 10).

The Apache-licensed Icebear source code is available at <https://github.com/Noble-Lab/icebear>.

8) Is it envisageable/meaningful to consider alternative VAE architectures for this task, e.g., using coupled VAEs, considering different species as different modalities, and the problem as a language translation problem?

The benefit of having one VAE is that we can conveniently learn a shared gene axis among orthologs across multiple species, and directly swap the species factor to predict any gene’s expression changes. We agree with the reviewer that loosening the orthology mapping criteria by coupling VAEs trained from different species could allow more flexibility in model training. We have added this idea to the discussion section as follows:

Currently, Icebear is designed to model cross-species gene expression using one-to-one orthology relationships. This approach discards a subset of duplicated genes that potentially harbor valuable information about cellular function and evolution. In this study, we observe that the advantage of Icebear’s cross-species alignment performance against SATURN shrinks as the median cell UMI decreases (Supplementary Figure 1). In the future, inspired by ideas from SATURN [1] and coupled variational autoencoders [5], we could extend Icebear to include all genes, rather than only one-to-one orthologs. In principle, such an extension to Icebear could enhance the cross-species translation performance without losing information from non-orthologous genes.

Some minor points:

1) Is the median sequencing depth of 450 UMIs considered enough for studying single-cell level expression of over 10,000 genes?

While higher UMI is more desirable, experiments are subjected to cost limitations and the balance between cell quantity and UMI coverage. It is not unusual for single-cell datasets to have a median UMI in the level of several hundreds. For example, in the cell atlas dataset used in the paper (Han et al. Cell 2018 and Han et al. Nature 2020), mouse cells have a median UMI of 688 out of 18606 genes; meanwhile, human cells have a median UMI of 591 out of 16750 genes. In our sci-RNA-seq dataset, the median UMI of 450 is calculated based on 9878 ortholog-matched genes, which is no sparser than the cell atlas dataset. In the scenario where cells are not deeply sequenced, Icebear can be particularly helpful to take as input the sparse measurement across many cells to impute cellular profiles. With the increasing experimental efforts to capture more cells for comprehensive characterization of tissues and organs at a limited cost, we anticipate that Icebear will be able to integrate and attribute a wide range of single-cell profiles across species.

2) The sentence “This process yielded 10,030 genes with orthologs shared across the three species, 70 of which were added through transitive relationships,” is repeated twice.

We thank the reviewer for catching this typo, which has been fixed.

3) The definition of the “indiv predictions” baseline remains unclear to me (what “the mean of similarity measurements between our predictions with each donor in the test set” exactly means). Overall, the description of the baselines lacks clarity; better to have more formal definitions.

The “indiv predictions” baseline represents the mean similarity across donors within the held-out test set. Specifically, we calculated the Pearson correlation of sequencing-depth normalized and log-transformed pseudobulk cell-type specific profiles between each pair of donors. We then reported the mean correlation across all donor pairs as the “indiv predictions” baseline. We have added to the mansucript more detail about all of the baselines:

1. Donor baseline (“donor baseline”): a “cheating” baseline that calculates the mean similarity across donors within the held-out test set. Here, we calculate the Pearson correlation of sequencing-depth normalized and log-transformed pseudobulk cell-type specific profiles between each pair of donors and report the mean correlation across all donor pairs.

2. Species baseline (“species baseline”): similarity between the original held-out cell type’s profile in human and the corresponding cell type’s profile in mouse. Genes are transferred to the target species through ortholog mapping and Pearson correlation between human and mouse sequencing-depth normalized and log-transformed pseudobulk cell-type specific profiles are reported.

3. Celltype baseline (“celltype baseline”): a “lazy” but close baseline that calculates the similarity between profiles of the held-out cell type and a similar cell type that has measurements in the target species. We first identify the cell type that has the most similar pseudobulk gene expression profile to the held-out cell type in the source species, calculated based on Euclidean distance on the sequencing-depth-normalized and log-transformed pseudobulk profiles. This baseline is created to ensure that our prediction captures more meaningful signals of the held-out cell type compared to guesses using a similar existing one.

4) Figure 2D is not informative and it is not clear how much it adds to the main text compared to Figure 2C. I suggest moving it to the supplementary materials.

We thank the reviewer for this suggestion. Figure 2D is added to the main figure to emphasize that Icebear can accurately perform cross-species prediction at very fine-scaled cell-type resolutions. Together with Figure 2C, it demonstrates that Icebear can generalize to different cell type resolutions. We have opted to leave it in the main text for now, but are open to the idea of moving it if necessary to save space.

5) What is Oligo-OPC? Any insight into why IceBear works worst for this cell type?

We have now clarified in the manuscript that Oligo-OPC is a cell type category, including Oligodendrocytes and Oligodendrocyte progenitor cells. There are several possible reasons why Icebear performs worst in this cell type. First, compared to neurons, there are fewer cell types similar to Oligo-OPC in human for the model to borrow information from. Second, because Icebear learns a general cross-species projection pattern based on all other cell types, cell types with poor cross-species prediction performance are likely those that have acquired cell-type-specific evolutionary patterns. For more details, please refer to the discussion section:

“Interestingly, we observed that Icebear’s prediction accuracy varies by cell type. For example, the accuracy in predicting expression values of human non-neuronal cells on the M1 dataset tends to be lower than that of other cell types. There could be two reasons for this variability. First, previous studies have found that glia cells are more evolutionarily diverged [12], so a general cross-species algorithm trained on cell types other than astrocytes may not accurately capture astrocyte-specific variations. Cells with immune functions, such as microglia, may be especially variable among species due to different exposure to pathogens. The other possible reason is that neuronal cell types are more abundant and their profiles and functions are more cohesive than those of glial cell types, which could lead to an easier machine learning task. In line with this explanation, we observed that housekeeping genes are more accurately predicted across species, presumably because housekeeping genes tend to be functionally conserved across species and cell types and thus are more easily captured by Icebear’s projection framework. In the future, a closer look at the genes whose predicted expression profiles are very different from their observed profiles could potentially reveal genes that adapt to the species-specific environment in a cell-type-specific manner. ”

6) I lack an understanding of what the term “translation algorithm” means in the phrase “may not only follow a general cross-species translation algorithm.”

We have clarified that in the text by changing the phrase “translation algorithm” to “expression pattern shift”. The full sentence now is changed to:

Because Icebear performs non-linear projection of cellular profiles across species, we hypothesize that a gene with large cell-type specific functional divergence during evolution may participate in species- and cell-type-specific adaptation processes, and thus may not follow a general crossspecies expression pattern shift.

7) Figure 2: “profiles in missing major cell types in the human motor cortex”: what does “missing” mean here? Held out?

The cell types held out from training. We have clarified that in the legend of Figure 2C accordingly.

(C) Barplot showing Pearson correlation coefficient (cor) between predicted and observed gene expression profiles in the “held-out” major cell types in human motor cortex. For each cell type, the Pearson correlation coefficient is compared between Icebear’s prediction (dark green bar) and two baselines (species and celltype). We also compared “indiv donorbaseline” (correlation between individual donors) with Icebear’s correlation with individual donors (“indiv prediction”, light green bar).

8) Multiple unclear formulations: just a couple of examples: “log2 fold change of genes across species,” “asking whether gene expression increases from halved gene dose when autosomal genes become X-linked and only have a single copy in XY males during mammalian sex chromosome evolution,” etc.

We thank the reviewer for the feedback and have corrected the ambiguity in the main text as follows.

(D) Boxplot of gene expression log2 fold changes from the lower to the upper species. Genes are grouped by their X-linked pattern. We are particularly interested in asking when autosomal genes become X-linked and have halved copy number in XY males during mammalian sex chromosome evolution, whether their gene expression increases from halved gene dose to compensate for this copy number changes.

9) Figures 4A and 4B—comparing them by visual inspection is not informative (especially considering the point sizes). Can this be quantified?

We thank the reviewer for this suggestion; we have quantified the species mixing using the LISI score and showed that Icebear can bring single cells across different species closer to a shared embedding space from the original measurement (Figure 10 in this document).

Compared to the original measurement, Icebear is able to bring mouse and human cells closer to a shared space, as measured by the LISI score (Supplementary Figure 10).

Figure 10: Quantification of cross-species integration on the sci-RNA-seq dataset LISI scores calculated based on Icebear’s embeddings or the PCA embeddings of the original measurements. For each tissue (i.e., brain or heart), we calculate how well cells from different species are mixed together. 50,000 cells are randomly sampled from the cell population ten times, and each dot represents the LISI score on one random sampling.

References

[1] Yanay Rosen, Maria Brbi´c, Yusuf Roohani, Kyle Swanson, Ziang Li, and Jure Leskovec. Toward universal cell embeddings: integrating single-cell rna-seq datasets across species with saturn. Nature Methods, pages 1–9, 2024.

[2] T. E. Bakken, N. L. Jorstad, Q. Hu, B. B. Lake, W. Tian, B. E. Kalmbach, H. Crow, R. D. Hodge, F. M. Krienen, S. A. Sorensen, et al. Comparative cellular analysis of motor cortex in human, marmoset and mouse. Nature, 598(7879):111–119, 2021.

[3] X. Han, Z. Zhou, L. Fei, H. Sun, R. Wang, Y. Chen, H. Chen, J. Wang, H. Tang, W. Ge, et al. Construction of a human cell landscape at single-cell level. Nature, 581(7808):303–309, 2020.

[4] X. Han, R. Wang, Y. Zhou, L. Fei, H. Sun, S. Lai, A. Saadatpour, Z. Zhou, H. Chen, F. Ye, et al. Mapping the mouse cell atlas by microwell-seq. Cell, 172(5):1091–1107, 2018.

[5] R. Zhang, Laetitia L. Meng-Papaxanthos, J. Vert, and W. S. Noble. Semi-supervised single-cell crossmodality translation using polarbear. In International Conference on Research in Computational Molecular Biology, pages 20–35. Springer, 2022.

[6] The Tabula Sapiens Consortium*, Robert C Jones, Jim Karkanias, Mark A Krasnow, Angela Oliveira Pisco, Stephen R Quake, Julia Salzman, Nir Yosef, Bryan Bulthaup, Phillip Brown, et al. The tabula sapiens: A multiple-organ, single-cell transcriptomic atlas of humans. Science, 376(6594):eabl4896, 2022.

[7] Yuan Liao, Lifeng Ma, Qile Guo, Weigao E, Xing Fang, Lei Yang, Fanwei Ruan, Jingjing Wang, Peijing Zhang, Zhongyi Sun, et al. Cell landscape of larval and adult xenopus laevis at single-cell resolution. nature communications, 13(1):4306, 2022.

[8] Mengmeng Jiang, Yanyu Xiao, Weigao E, Lifeng Ma, Jingjing Wang, Haide Chen, Ce Gao, Yuan Liao, Qile Guo, Jinrong Peng, et al. Characterization of the zebrafish cell landscape at single-cell resolution. Frontiers in cell and developmental biology, 9:743421, 2021.

[9] R. Lopez, J. Regier, M. B. Cole, M. I. Jordan, and N. Yosef. Deep generative modeling for single-cell transcriptomics. Nature Methods, 15(12):1053–1058, 2018.

[10] I. Korsunsky, N. Millard, J. Fan, K. Slowikowski, F. Zhang, K. Wei, Y. Baglaenko, M. Brenner, P. Loh, and S. Raychaudhuri. Fast, sensitive and accurate integration of single-cell data with Harmony. Nature Methods, 16:1289–1296, 2019.

[11] A. J. Tarashansky, J. M. Musser, M. Khariton, P. Li, D. Arendt, S. R. Quake, and B. Wang. Mapping single-cell atlases throughout metazoa unravels cell type evolution. Elife, 10:e66747, 2021.

[12] William G Pembroke, Christopher L Hartl, and Daniel H Geschwind. Evolutionary conservation and divergence of the human brain transcriptome. Genome biology, 22(1):1–33, 2021.

**2^nd^ round**

**Reviewer 1**

The authors have addressed all my previous concerns.

A typo: Pg 17, Line 27-28, Supplementary figure number is not available.

**Reviewer 2**

I am satisfied with the revisions made to the manuscript, incorporating the suggestions from both reviewers. My only remaining recommendation is to update the provided GitHub repository with the following:
1) The trained model weights used for the analysis that generated the manuscript figures.
2) Jupyter notebooks demonstrating how to load these model weights and perform the key tasks outlined in the manuscript (eg, predicting gene expression changes)
3) A more detailed README that includes instructions on how to access the datasets used in the manuscript, along with, if possible, the code for the preprocessing steps.

**Authors’ response**
